# Characterization of the association and sequestration of RNA-binding proteins by single-stranded DNA chimera: An ssDNA method for RBPs

**DOI:** 10.3724/abbs.2024157

**Published:** 2024-09-26

**Authors:** Jianyang Wang, Wenliang Guan, Leilei Jiang, Hongyu Hu

**Affiliations:** 1 Key Laboratory of RNA Innovation Science and Engineering Shanghai Institute of Biochemistry and Cell Biology Center for Excellence in Molecular Cell Science Chinese Academy of Sciences Shanghai 200031 China; 2 Fujian Key Laboratory of Innate Immune Biology Biomedical Research Center of South China College of Life Science Fujian Normal University Fuzhou 350117 China; 3 University of Chinese Academy of Sciences Beijing 100049 China

The biomolecular assemblies (condensates or aggregates) formed by mutant proteins are a pathological hallmark of neurodegenerative diseases [ 1, 2]. Some RNA-binding proteins (RBPs) are typically prone to aggregation that is closely associated with disease pathologies [3]. These RBPs include numerous well-recognized pathogenic proteins [ 4, 5], such as TAR DNA binding protein of 43 kDa (TDP-43), fused in sarcoma (FUS), ataxin-2 (Atx2), and poly(A)-binding protein nuclear 1 (PABPN1). Recent studies have revealed that liquid-liquid phase separation (LLPS), as a mechanism, underlies the highly dynamic and reversible granule formation of RBPs [4], and highlighted that multivalent RNA molecules play crucial roles in this process. These granules are necessary for diverse physiological functions, such as RNA splicing, trafficking, and even RNA storage, during stress [5]. However, the aberrant phase transition of these mutant RBPs usually results in the formation of solid-like aggregates or inclusions within both the cytoplasm and nucleus. More importantly, aggregates formed by RBPs can sequester specific proteins, RNAs or other interacting partners, consequently contributing to RBP-related pathologies [1]. For example, wild-type PABPN1 forms dynamic nuclear speckles with the assistance of poly(A) RNAs, whereas Ala expansion of PABPN1 results in the formation of aggregates, which are involved in the disease progression of oculopharyngeal muscular dystrophy (OPMD). Although the biological importance of various RBP granules is realized in either the cytoplasm or nucleus, how RNA regulates the formation of granules and the transition to aberrant RBP aggregates remains largely unknown.

The interaction of a protein with other biomolecules (proteins, nucleic acids, *etc*.) is the prerequisite for the protein executing its normal biological function in cells. Identifying protein-protein and protein-RNA interactions is fundamental for the biochemical investigation of an individual protein and for attempts to understand the functional role of the protein. To date, many methods for studying protein-protein interactions have been developed on the basis of various principles, but it is still difficult to clarify whether the interactions between proteins, especially the RBPs involved, are direct or indirect, since RBPs generally bind to diverse RNAs closely and are incorporated into macromolecular ribonucleoprotein (RNP) complexes.

We have taken several pairs of RBPs as examples, including TDP-35 (C-terminal 35-kDa fragment of TDP-43) with TDP-43 or TIA1 [6], PABPN1 with a 25-kDa component of the mammalian cleavage factor I complex (CFIm25) [7] and Atx2 with DEAD-box RNA helicase 6 (DDX6) [8], and applied modified co-immunoprecipitation (Co-IP) and supernatant/pellet (S/P) fractionation experiments to characterize the association and sequestration of RBPs by using single-stranded DNA (ssDNA) chimera under ribonuclease (RNase) treatment. We designed several pieces of ssDNA oligonucleotides to mimic particular RNAs in cells that may mediate the association and sequestration of RBPs. The association of RBP proteins generally requires binding with multivalent RNA chains, since the bound RNAs tend to incorporate into a large protein-RNA complex with the help of RNA molecules. In Co-IP assay, especially for RBPs, RNase is often utilized to digest RNA in cell lysates to characterize whether the association of different RBPs is direct or indirect [6]. It is important for us to demonstrate the active role of particular RNAs in the association or interaction of RBPs. Therefore, we designed and synthesized ssDNA chimeras to mimic the corresponding RNA that specifically bind to both RBPs simultaneously. In this case, ssDNA is used for rescuing the association of RBPs under the condition of RNase treatment, since the ssDNA oligonucleotide is resistant to nuclease activity.

**Figure 1:**
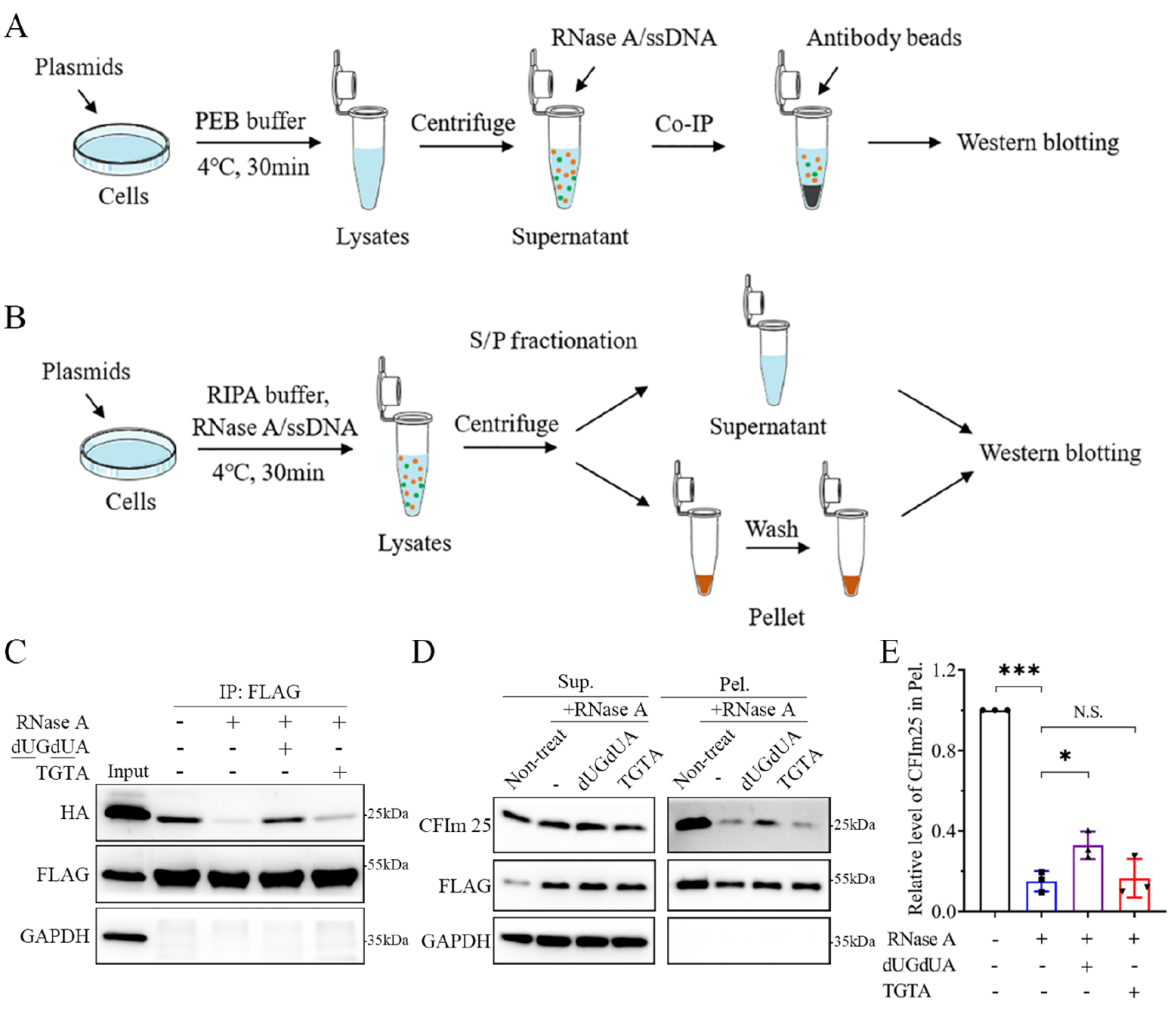
Figure 1 Characterization of the association and sequestration of RBPs by designer ssDNA chimera (A,B) Schematic diagrams of the modified co-IP assay (A) and S/P fractionation experiment (B). (C) Characterization of the association between PABPN1 and CFIm25. HEK293T cells were co-transfected with FLAG-PABPN1 and HA-CFIm25, and 48 h later, the cell lysates were subjected to a co-IP assay with an anti-FLAG antibody and western blot analysis. RNase A and/or the indicated ssDNA were added to the cell lysates before immunoprecipitation. (D) Characterization of the sequestration of endogenous CFIm25 by PABPN1-24A. HEK293T cells were transfected with FLAG-PABPN1-24A, and then, RNase A and/or the indicated ssDNA (∼5 μM) were added to the cell lysates, which were subjected to S/P fractionation with western blot analysis. PABPN1-24A was detected with an anti-FLAG antibody, while endogenous CFIm25 was detected with an anti-CFIm25 antibody. Non-treat, without RNase treatment; dUA dUA, the dUAdUA ssDNA that specifically binds with PABPN1 and CFIm25; TGTA, TGTA ssDNA, dU was replaced with a T base; Sup., supernatant; Pel., pellet. (E) Quantification of the relative CFIm25 level in the pellet fraction in (D). Data are shown as the mean ± SD (n = 3). *P < 0.05, ***P < 0.001. N.S., no significance.

To design ssDNA chimeras for the RBPs of interest, first, the RNA sequences that bind to the two RPBs should be defined. The ssDNA should contain at least two portions (motifs) that specifically bind to each RBP, and each ssDNA portion may include 2–3 repeats of the binding sequence, so that the ssDNA can be recognized and bound efficiently by each RBP. Notably, the T base in ssDNA may sometimes be replaced with the U base (dU) for some more specific-binding RBPs, such as PABPN1 [7]. In the case of TDP-43 with Atx2, the binding specificities of the RNA sequences for TDP-43 [9] and Atx2 [10] are UG-rich and AUUUUU (AU _5_), respectively; then, the TG repeat portion is designed to bind to TDP-43, and the AT _5_ repeat is to bind to Atx2. Thus, an integrated method of co-IP and S/P fractionation was applied to characterize the association and sequestration of RBPs by combining ribonuclease (RNase) and ssDNA treatments ( Figure 1A,B). In this case, an ssDNA chimera of particular sequence ( Table 1) was included in the cell lysates to mimic the RBP-bound RNAs, and then a modified co-IP assay was applied to characterize whether the RNA-assisted association between RBPs is direct or indirect. To investigate whether RBP assemblies (condensates or aggregates) sequester their interacting partners via particular RNAs, a modified S/P fractionation experiment was performed to detect the sequestered proteins and RNA specificity.

**Table:** **Table 1** Nucleotide sequences of ssDNA applied in this study

Nucleotide	Sequence	Note
dUGdUA	GGGdUGdUAAACAGA dUGAdUGdUAdU AAAAAAAAAAAAAAAAAAAAAAAAAAAAAAAAAAAAAA	dUGdUA repeat and poly(A), for CFIm25 and PABPN1
TGTA	GGGTGTAAACAGATGATGTATAAAAAAAAAAAAAAAAAAAAAAAAAAAAAAAAAAAAAA	TGTA repeat and poly(A), as a control for dUGdU A
(TG) _29_	TGTGTGTGTGTGTGTGTGTGTGTGTGTGTGTGTGTGTGTGTGTGTGTGTGTGTGTGTG	TG repeat, for TDP-43/TDP-35
(AT _5_) _5_	ATTTTTATTTTTATTTTTATTTTTATTTTT	ATTTTT repeat, for Atx2
(TG) _24_+(AT _5_) _5_	TGTGTGTGTGTGTGTGTGTGTGTGTGTGTGTGTGTGTGTGTGTGTGTGATTTTTATTTTTATTTTTATTTTTATTTTT	TG repeat and ATTTTT repeat, for TDP-43/TDP-35 and Atx2

Our previous study indicated that PABPN1-24A (a variant with 24 alanines) could form solid aggregates and sequester CFIm25, a component of 3′-UTR processing complex [7]. We further demonstrated the role of particular RNAs in the association and sequestration processes by using the ssDNA method ( Figure 1C‒E). First, the modified co-IP assay confirmed that wild-type PABPN1 (with 10 alanines) associated with CFIm25 and their association was almost disrupted upon the addition of RNase A. dUGdUA ssDNA largely restored the decreasing effect of RNase-A treatment, whereas TGTA as a control could not ( Figure 1C), suggesting that the association between PABPN1 and CFIm25 is dependent on specific RNA sequences. Analogously, S/P fractionation revealed that sequestration of endogenous CFIm25 by PABPN1-24A aggregates was mediated by some particular RNAs, in which the insoluble fraction of CFIm25 was significantly decreased upon RNase-A treatment and restored by the addition of dUGdUA but not TGTA ( Figure 1D,E). Interestingly, although the binding motif for CFIm25 is the UGUA sequence [11], the TGTA ssDNA is not capable of assisting in the association and sequestration of CFIm25, suggesting that the U bases in the dUGdUA sequence may play a critical role in these processes [7].

**Figure 2:**
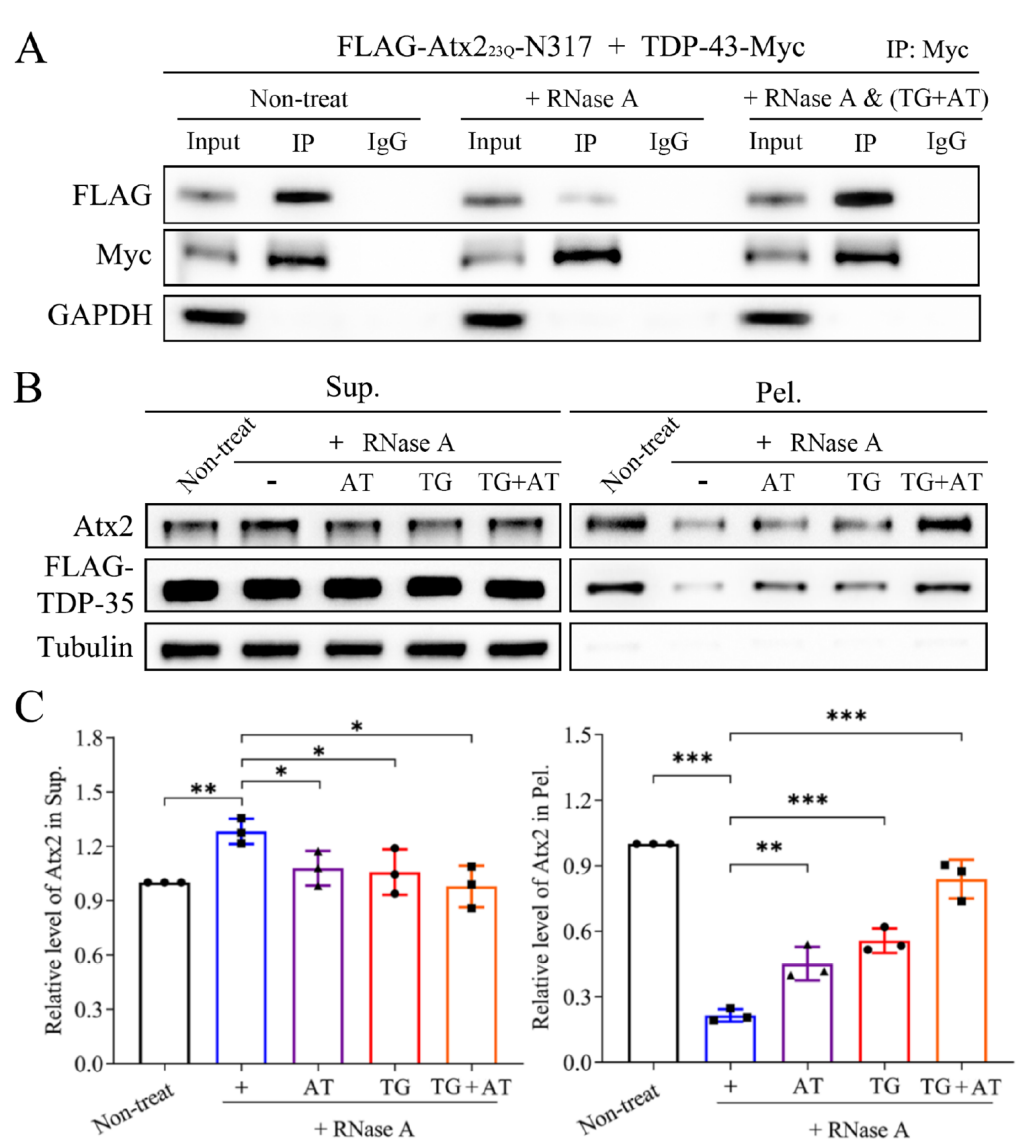
Figure 2 Elucidating the roles of particular RNAs in the association and sequestration of TDP-43 and Atx2 by ssDNA method (A) Co-IP assay for characterizing the effects of RNase A and/or ssDNA treatment on the association of TDP-43 with Atx2-N317. Atx223Q-N317, N-terminal 317-residue fragment of Atx2 with 23 glutamines. (B) S/P fractionation for examining the effects of RNase A and/or ssDNA treatment on the sequestration of endogenous Atx2 by TDP-35. (C) Quantification of the relative levels of endogenous Atx2 (B) in the supernatant and pellet fractions. AT, (AT5)5; TG, (TG)29; TG + AT, (TG)24 + (AT5)5. Sup., supernatant; Pel., pellet. Data are shown as the mean ± SD (n = 3). *P < 0.05, **P < 0.01, ***P < 0.001.

TDP-43 can associate with and sequester numerous RBPs (such as TIA1), and their association and sequestration are assisted by RNA binding [6], while Atx2 is also responsible for RNA metabolism via binding with other RBPs on the basis of its N-terminal LSm and LSmAD domains [ 10, 12]. We presumed that the association and sequestration between TDP-43 and Atx2 are mediated by particular RNA sequences. To examine the role of RNA in the association of TDP-43 with Atx2, we co-transfected TDP-43-Myc and FLAG-Atx2 _23Q_-N317 (N-terminal 317-residue fragment of Atx2) [8] in HEK293T cells and treated the cell lysates with RNase A and/or ssDNA, followed by a co-IP assay as described previously [ 6, 7]. In the absence of RNase A, a clear band corresponding to the Atx2-N317 protein was clearly observed in the gel, indicating that TDP-43 could associate with Atx2-N317 in cells ( Figure 2A). When the cell lysates were treated with RNase A, the Atx2-N317 band was markedly attenuated. However, this band could be recovered considerably by the addition of an ssDNA, namely, (TG) _24_ + (AT _5_) _5_ ( Table 1 and Figure 2A), indicating that the association between TDP-43 and Atx2 is mediated by TG- and AU-rich RNAs. Thus, TDP-43 indirectly interacts with Atx2, and some particular RNAs play important roles in their association. This protein-RNA complex might take part in the sequestration of other RBPs, leading to formation of the RNP complex in a condensed state.

Our previous studies demonstrated that TDP-35 can form aggregates (or inclusions) in the cytoplasm and sequester TDP-43 into cytoplasmic inclusions through binding with RNA [6]. To characterize the role of RNA in mediating sequestration of cytoplasmic Atx2, we used TDP-35 as an example and performed S/P fractionation experiments under the conditions of RNase and/or ssDNA treatment. The results showed that RNase-A treatment significantly disrupted the sequestration of Atx2 by TDP-35 aggregates. The single-motif ssDNA, either (AT _5_ ) _5_ or (TG) _29_, could partially recover the disrupting effect of RNase-A digestion, while the soluble amount in the supernatant was reduced slightly ( Figure 2B,C). Intriguingly, the chimeric ssDNA (TG) _24_ + (AT _5_) _5_ remarkably recovered the sequestration effect. This finding implies that sequestration of Atx2 by TDP-35 aggregates is dependent on their association, which is mediated by particular RNA sequences.

In summary, we developed an integrated method to elucidate the roles of RNA molecules in the association and sequestration of RBPs by using an ssDNA chimera, which demonstrated that some particular RNAs in cells not only participate in the association of RBPs but also mediate the sequestration of their interacting partners. The designer chimeric ssDNA corresponding to the specific RNA sequences that bind to RBPs is applicable for studying RNP assembly and RBP sequestration related to disease pathology.
